# Vitamin D Receptor Gene Ablation in the Conceptus Has Limited Effects on Placental Morphology, Function and Pregnancy Outcome

**DOI:** 10.1371/journal.pone.0131287

**Published:** 2015-06-29

**Authors:** Rebecca L. Wilson, Sam Buckberry, Fleur Spronk, Jessica A. Laurence, Shalem Leemaqz, Sean O’Leary, Tina Bianco-Miotto, Jing Du, Paul H. Anderson, Claire T. Roberts

**Affiliations:** 1 Robinson Research Institute, Adelaide, Australia; 2 School of Paediatrics and Reproductive Health, University of Adelaide, Adelaide, Australia; 3 School of Agriculture, Food and Wine, University of Adelaide, Adelaide, Australia; 4 School of Pharmacy and Medical Sciences, Division of Health Sciences, University of South Australia, Adelaide, Australia; 5 Shanghai Institute of Planned Parenthood Research, Shanghai, China; University of Alabama at Birmingham, UNITED STATES

## Abstract

Vitamin D deficiency has been implicated in the pathogenesis of several pregnancy complications attributed to impaired or abnormal placental function, but there are few clues indicating the mechanistic role of vitamin D in their pathogenesis. To further understand the role of vitamin D receptor (VDR)-mediated activity in placental function, we used heterozygous *Vdr* ablated C57Bl6 mice to assess fetal growth, morphological parameters and global gene expression in *Vdr* null placentae. Twelve *Vdr* ^+/-^ dams were mated at 10–12 weeks of age with *Vdr* ^+/-^ males. At day 18.5 of the 19.5 day gestation in our colony, females were euthanised and placental and fetal samples were collected, weighed and subsequently genotyped as either *Vdr* ^+/+^, *Vdr* ^+/-^ *or Vdr* ^-/-^. Morphological assessment of placentae using immunohistochemistry was performed and RNA was extracted and subject to microarray analysis. This revealed 25 genes that were significantly differentially expressed between *Vdr* ^+/+^ and *Vdr* ^-/-^ placentae. The greatest difference was a 6.47-fold change in expression of *Cyp24a1* which was significantly lower in the *Vdr* ^-/-^ placentae (P<0.01). Other differentially expressed genes in *Vdr* ^-/-^ placentae included those involved in RNA modification (*Snord123*), autophagy (*Atg4b*), cytoskeletal modification (*Shroom4*), cell signalling (*Plscr1*, *Pex5*) and mammalian target of rapamycin (mTOR) signalling (*Deptor* and *Prr5*). Interrogation of the upstream sequence of differentially expressed genes identified that many contain putative vitamin D receptor elements (VDREs). Despite the gene expression differences, this did not contribute to any differences in overall placental morphology, nor was function affected as there was no difference in fetal growth as determined by fetal weight near term. Given our dams still expressed a functional VDR gene, our results suggest that cross-talk between the maternal decidua and the placenta, as well as maternal vitamin D status, may be more important in determining pregnancy outcome than conceptus expression of VDR.

## Introduction

Normal fetal development is undoubtedly underpinned by normal placental function. The placental vascular network provides an interface between the fetus and mother for the exchange of gases, nutrients and wastes [1]. Additionally, the placenta acts as an endocrine organ responsible for the production of numerous hormones which maintain pregnancy and orchestrate maternal adaptation to pregnancy [2]. Maternal nutrient status underlies the availability of nutrients being transferred to the fetus to support optimal growth. Placental research is increasingly focused on how the organ adapts to support adequate fetal growth in a potentially sub-optimal nutrient available environment [3].

The prevalence of vitamin D deficiency and insufficiency in pregnant women is increasing worldwide [4, 5] and accumulating evidence associates vitamin D deficiency with a range of pregnancy complications including preeclampsia [6, 7], gestational diabetes mellitus [8] and preterm birth [9]. Additionally, maternal vitamin D deficiency increases the chance of delivering a baby who is small for gestational age [10, 11] and has also been linked to the development of asthma [12], autism [13], and reduced bone mineral accrual [14, 15] in the offspring. During human pregnancies, serum levels of the active form of vitamin D_3_ (1,25(OH)_2_D_3_) increase by 2 to 5-fold [16–18] suggesting an important role for vitamin D in supporting the pregnancy and fetal development. While vitamin D supplementation has been reported to help neonatal outcomes [19], the lack of high quality intervention data to confirm a causal role for vitamin D in pregnancy outcomes [20] and a description of the underlying mechanisms are lacking.

While the secosteroid hormone, 1,25(OH)_2_D_3_, is widely associated with calcium and phosphate homeostasis [21], other functions for 1,25(OH)_2_D_3_ activity have been identified such as in modulation of immune [22] and vascular [23] function, brain [24] and muscle [25] development, and bone remodelling [26–28]. In general, the actions of 1,25(OH)_2_D_3_, are broadly associated with regulating cell proliferation and differentiation [29, 30]. The effects of 1,25(OH)_2_D_3_ are mediated through the vitamin D receptor (VDR), a predominantly nuclear receptor, expressed in numerous tissues including the placenta [31, 32]. The liganded VDR, together with retinoid X receptor (RXR) in a dimer complex, binds to genomic vitamin D responsive elements (VDREs), located primarily in upstream flanking regions of genes, and recruit a cell-specific transcription factor complex which regulates the expression of numerous genes [33, 34]. Local synthesis and metabolism of 1,25(OH)_2_D_3_ within the placenta is likely to occur given that placental cells expresses both *CYP27B1*, which encodes the enzyme to produce 1,25(OH)_2_D_3_, and *CYP24A1*, which encodes for enzyme responsible for the catabolism of 1,25(OH)_2_D_3_ [35].

Although vitamin D activity in the decidua is suggested to regulate immune tolerance during pregnancy [22], the evidence that supports the link between VDR expression, placental growth, function and fetal outcome is lacking. Previously, *Vdr* knockout (*Vdr* ^*-/-*^) dams have been shown to exhibit both a reduction in the rate of conception and reduced fetal weights when compared to heterozygous (*Vdr* ^*+/-*^) dams [36]. However, such studies are unable to discern whether the ablation of *Vdr* in the placenta contributes to these outcomes. Studies on vitamin D and placental function are limited and have focused on immune function within the maternal decidua of *Vdr* knockout mice [37] or on placental morphometry in dietary vitamin D restricted animals [38]. Thus, we used heterozygous matings of *Vdr* knockout mice to investigate the effects of *Vdr* ablation specifically in the conceptus by characterising placental morphology, fetal growth and global placental gene expression measures near term. The study design specifically excluded confounding effects of perturbed *Vdr* signalling in the mother to elucidate placenta specific effects. We chose late gestation as a first step in elucidating the role of vitamin D signalling in placental structural and functional development as this corresponds most closely to the time at which placentas could be sampled from women.

## Methods

### Animals

Ethics approval was obtained from both the SA Pathology/Central Northern Adelaide Health Service Animal Ethics Committee and the University of Adelaide Animal Ethics Committees with all animal work complying with the Australian Code of Practice for the Care and Use of Animals. Global *Vdr* ablated C57Bl6 mice (strain B6.129S4-VDRtm1Mbd/J, Jackson Laboratory JAX Mice Services) were generated as previously described [39]. At weaning, 12 virgin *Vdr* ^+/-^ females were fed a standard rodent diet containing 0.8% calcium and 0.7% phosphorus (Specialty Feeds), water *ad libitum* and were maintained on a 12:12 light-dark cycle. Females at 10–12 weeks of age were mated with a *Vdr* ^+/-^ male to generate offspring of all three genotypes (*Vdr* ^-/-^, *Vdr* ^+/-^, *Vdr* ^+/+^). The day of copulatory plug detection was designated day 0.5 of pregnancy. On day 18.5 of the 19.5 day pregnancy in our colony, females were anaesthetised with an intraperitoneal injection of Avertin (20 mg/mL) to collect blood and then killed via cervical dislocation. Fetuses and placentae were collected and weighed. The placentae were bisected mid-sagittally with half stored RNAlater and subsequently at -80°C for gene expression analyses, while the remaining half was fixed for histological analyses.

### Genotyping

To determine *Vdr* genotype and fetal sex, DNA was extracted from fetal tails using the salting-out procedure detailed in [40]. Following DNA quantification, samples were diluted to 20 ng/μL in TE buffer and used in PCR for *Vdr* genotyping (Table A in S1 File) [41] or *Sry* detection (Table B in S1 File) [42], respectively. Final PCR reactions were performed on 10 ng/μL of DNA in a 20 μL reaction containing 10 μL SsoFast EvaGreen Supermix (BioRad) and 10 μM *Vdr* primers or 200 nM *Sry* primers. Outcomes of the PCR were validated using gel electrophoresis on a 2% and 2.7% agarose gel for *Vdr* and *Sry*, respectively (Fig A in S1 File).

### Placental histology

Histological analyses were performed on all placentae from *Vdr* ^+/-^ dams to capture all genotypes. Bisected placentae were washed twice in PBS over 2 hours to remove RNAlater then fixed in 10% neutral buffered formalin (Australian Biostain). Samples were subsequently washed in three changes of PBS and stored in 70% ethanol prior to paraffin embedding. 5 μm thickness full-face sections were stained with Masson’s Trichrome following standard protocols in order to determine mid sagittal labyrinth and junctional zone cross sectional areas or subjected to immunohistochemistry (IHC) as previously described [43].

Fetal capillaries and trophoblast cells in the placental labyrinth were identified by double-label IHC with anti-vimentin (#M7020, Dako, Agilent Technologies; 1/5 dilution) and anti-cytokeratin antibodies (#MAB3412, Merck Millipore; 1/100 dilution), respectively [43]. Briefly, antigen retrieval was performed with 0.3 mg/mL Pronase (P8811, Sigma-Aldrich) in PBS, with a Mouse-on-Mouse IHC kit (Abcam) used to prevent non-specific binding. Chromogen diaminobenzidine (DAB, Sigma Aldrich) was used to form a brown precipitate for anti-cytokeratin labelling and with 2% nickel II sulphate (Sigma Aldrich) to form a black precipitate for anti-vimentin labelling. Sections were counterstained with haematoxylin and eosin (Sigma Aldrich).

Immunohistochemically labelled slides were analysed by point counting ten fields per placenta to estimate volume densities and volumes of fetal capillaries, trophoblasts and maternal blood space and intercept counting to estimate the surface density and thickness of trophoblast for exchange, previously described in [43]. The coefficient of variation was <5%.

### RNA extraction, microarray preparation and qPCR

Placental tissue was homogenised using a Powerlyzer with ceramic 1.4 mm beads (Mo Bio Laboratories, Inc) before total RNA was extracted using Trizol (Invitrogen) following the manufacturer’s instructions. RNA integrity was determined using the Experion (BioRad) system.

For microarray, eight *Vdr* ^+/+^ and eight *Vdr* ^-/-^ placentae from six heterozygous dams were analysed. Biotinylated cRNA was prepared according to the standard Affymetrix protocol from 250 ng total RNA following the Manual Target Preparation Guidelines for GeneChip Whole Transcript (WT) Expression Arrays. RNA with RQI > 9 was sent to the Ramaciotti Centre for Genomics, Sydney, Australia, where 3.5 μg of fragmented and labelled single-stranded cRNA was hybridised on Affymetrix MoGene 2.1 ST arrays and washed and stained following the Manual Target Preparation Guidelines for GeneChip Whole Transcript (WT) Expression Arrays. Arrays were scanned using the Affymetrix GeneChip scanner.

For microarray validation, extracted RNA from 17 *Vdr* ^+/+^ and 16 *Vdr* ^-/-^ placentae was DNase treated using TURBO DNA-free (Ambion) as per the manufacturer’s instructions. PCR and subsequent agarose gel confirmed the absence of genomic DNA (Table C in S1 File). 500 ng of each sample was then reverse transcribed using the iScript cDNA Synthesis Kit (Bio-Rad). Each cDNA sample was diluted 1:20 before performing quantitative PCR (qPCR) in triplicate by real time PCR using TaqMan Gene Expression assays (Table D in S1 File). All qPCR gene expression data were normalised to *Hbms*.

### Microarray differential expression

Affymetrix Mouse Gene 2.1 ST array data were pre-processed, background subtracted and quantile normalised using the RMA method in the *Oligo* package. Array probes were annotated using the Bioconductor *Affymetrix mogene21* annotation data package, with all unannotated probes subsequently removed from the dataset. Testing for differential expression between groups was performed using linear models and Empirical Bayes methods, with contrasts between groups incorporating the mother as a blocking factor using the *Limma* package [44]. All *P*-values were corrected for multiple testing by calculating the false discovery rate (FDR). Microarray data have been deposited to NCBI GEO under accession GSE61583 and analysis code is included with the S2 File. Data analyses were performed in R version 3.1.1.

### VDRE enrichment analysis

The top up- and down-regulated genes between *Vdr* ^-/-^ and *Vdr* ^+/+^ placentae as determined in the microarray (>1.5 fold-change, p < 0.01) were analysed for presence of putative vitamin D responsive elements (VDRE’s) that potentially bind the RXRA::VDR transcription factor complex using oPOSSUM and the JASPAR vertebrate core profile for RXRA::VDR (MA0074.1) [45, 46]. For each gene, we searched for RXRA::VDR motifs in the 10 kb upstream and downstream sequences from the transcription start site using a conservation cut-off of 0.4, a matrix score threshold of 75% and a minimum specificity of 8-bits.

### Statistics

To test for morphological differences between *Vdr* genotypes, weighted mixed-effects linear models were fitted to the data and included fetal sex as a covariate and were weighted by litter size using the *lme* function in the *nlme* package in R v3.1.1. Gene expression differences were assessed by the Mann-Whitney test to calculate exact *P*-values. Results are reported as mean normalised expression ± standard error.

## Results

To examine the role of *Vdr* signaling in the placenta and the effects on fetal and placental growth and development, *Vdr* ^+/-^ females were mated with *Vdr* ^+/-^ males and sacrificed on day 18.5 of pregnancy. A standard Mendelian 1:2:1 ratio distribution of genotypes for *Vdr* was observed when accounting for and excluding resorptions. Of the 12 pregnancies, 77 fetuses were collected and analysed, with *Sry* genotyping revealing 45 female and 32 male fetuses (Table 1).

**Table 1:** Pregnancy characteristics of *Vdr* ^+/-^ dams at gestational day 18.5. Data expressed as mean ± SEM.

Parameter			*Vdr* ^+/-^ (*n = 12*)
Percentage conceived			100%
Weight gain during pregnancy (g)			18.13 ± 3.64
% Maternal weight gain during pregnancy			89.15 ± 19.89
Viable litter size			6.58 ± 0.54
Number reabsorptions			1.17 ± 0.32
Average Fetal Weight (g)			1.13 ± 0.016
Placental weight (g)			0.78 ± 0.074
	Male (*n = 34*)	Female (*n = 45*)	**P-value**
Fetal weight	1.16 ± 0.035	1.10 ± 0.019	0.008
Placental weight	0.12 ± 0.0029	0.11 ± 0.0033	0.255

### The effects of VDR depletion on fetal and placental parameters

**Fig 1:**
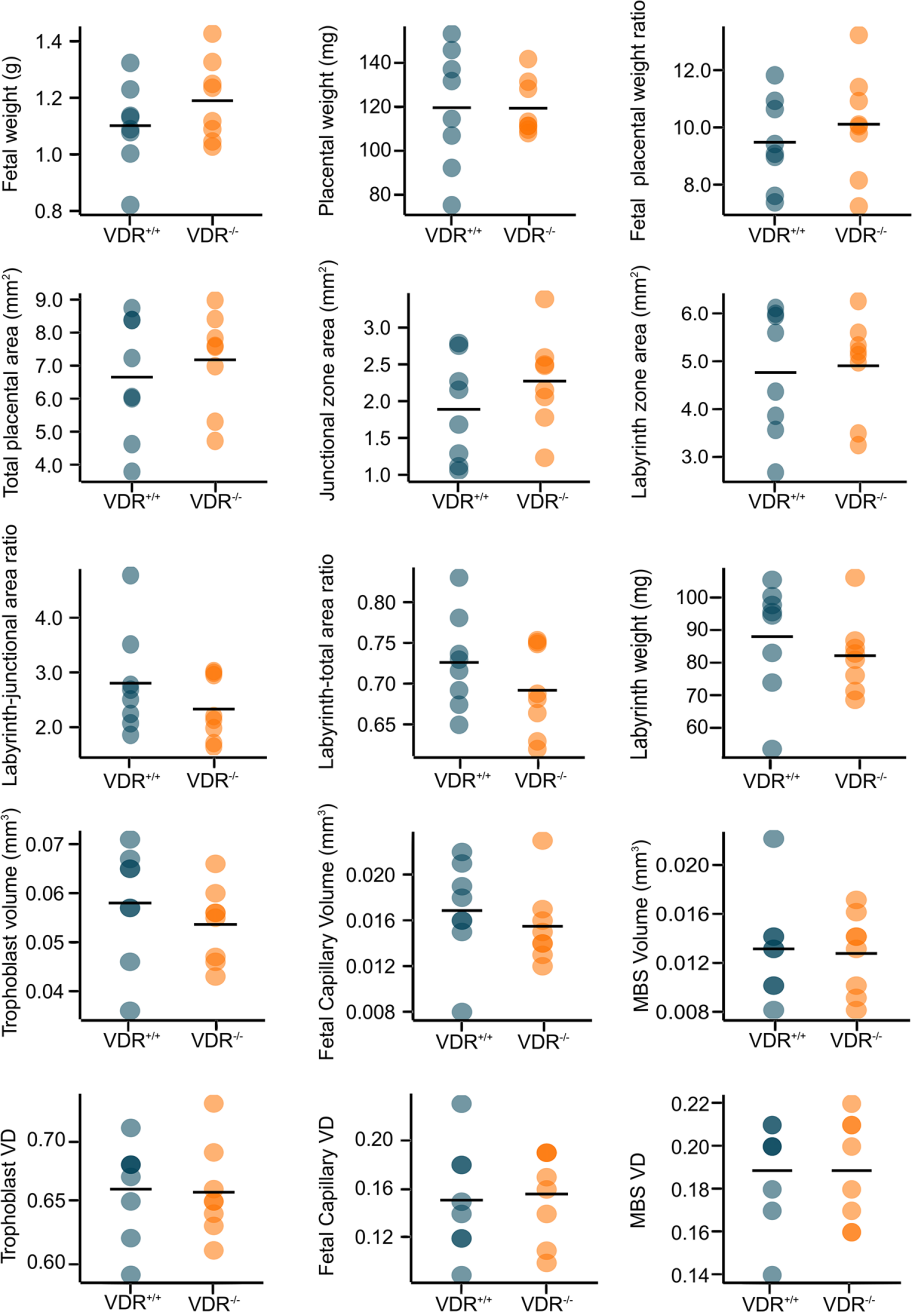
Comparison of mouse placental morphology measurements between *Vdr* ^-/-^ and *Vdr* ^+/+^ genotypes at day 18.5pc. No significant differences were observed in any of the morphology parameters assessed between the two genotypes (P>0.05) in the 8 *Vdr* ^***+/+***^ and 8 *Vdr* ^***-/-***^ placentas analysed by microarray. Horizontal line on each plot represents mean. MBS: maternal blood space; VD: volume density.

The effect of *Vdr* ablation on fetal and placental measures was assessed initially by analyzing fetal and placental weights in 17 *Vdr* ^+/+^, 54 *Vdr* ^+/-^ and 21 *Vdr* ^-/-^ conceptuses, with no significant differences detected across the genotypes (Fig 1 shows data for 8 *Vdr* ^+/+^ verses 8 *Vdr* ^-/-^ conceptuses for which microarray analyses were undertaken). Placental structure, examined firstly by Masson’s trichrome staining, revealed no significant differences in morphology between *Vdr* ^*-/-*^ and *Vdr* ^+/+^ genotypes. These morphology measures included total mid sagittal cross sectional area, junctional zone and labyrinth zone areas and the proportion of junctional zone to labyrinth zone. In mice, the placental labyrinth is the area in which physiological exchange of nutrients and waste products occurs between fetal and maternal bloodstreams, whereas the junctional zone contains placental stem cells and is involved in hormone production. A larger labyrinth or a higher labyrinth to junctional zone ratio suggests enhanced placental efficiency. Given there were no differences in the proportions of junctional and labyrinth zones, this suggests similar placental efficiency, which corresponds to the similar fetal weights across genotypes (Fig 1).

Further quantification of labyrinth zone structure using double-labelled IHC showed no significant differences between genotypes for volume densities or volumes of trophoblasts, fetal capillaries and maternal blood space, as well as surface density of trophoblast. Our data suggest feto-placental *Vdr* ablation does not affect placental composition nor functional capacity.

Altogether, analyses of fetal and placental parameters clearly indicated that there were no gross morphological differences that may underpin phenotypic changes such as hypocalcemia, hyperparathyroidism and rickets experienced by *Vdr* ^-/-^ pups from weaning [39, 47]. Such changes may however be modulated by placental or fetal gene expression differences.

### The effect of *VDR* ablation on the placental transcriptome

To test for the effect of *Vdr* ablation on gene expression in the placenta, transcriptome profiles of eight placentae per genotype were assessed by microarray. Twenty-five genes were detected as being differentially expressed between *Vdr* ^*-/-*^ and *Vdr* ^+/+^ placentae with an absolute fold change >1.3 and a false discovery rate (FDR) <0.05 (Table 2). The greatest difference was a 6.47-fold change (FDR = 0.0012) in the expression of *Cyp24a1*, which was lower in the *Vdr* ^*-/-*^ placentae. As *Cyp24a1* is directly upregulated through Vdr as part of the vitamin D metabolic pathway, severely reduced placental *Cyp24a1* expression in *Vdr* ^-/-^ placentae would be expected. Other genes that were differentially expressed included genes involved in RNA modification (*Snord123*), autophagy (*Atg4b*), cytoskeletal modification (*Shroom4*), cell signaling (*Plscr1*, *Pex5*, *Rgs17*), and mammalian target of rapamycin (mTOR) signaling (*Deptor*, *Prr5*). Of these differentially expressed genes, 12 were more highly expressed in *Vdr* ^-/-^ placentae and 13 had lower expression levels when compared to *Vdr* ^+/+^ placentae. No significant differences in gene expression between the sexes within each genotype were detected (data not shown).

**Table 2:** Genes differentially expressed between *Vdr* ^-/-^ and *Vdr* ^+/+^ placentae.

	Microarray				qPCR		
Gene	Fold change	*Vdr* ^*-/-*^ Expression	*P*-value	FDR	Fold change	*Vdr* ^*-/-*^ Expression	*P*-value
*Cyp24a1*	6.47	↓	5.0E-08	0.001	95.27	↓	<0.001
*Snord123*	1.58	↓	4.3E-06	0.027	-	-	-
*Atg4b*	1.33	↑	4.5E-06	0.027	-	-	-
*Snora28*	1.49	↓	5.5E-06	0.027	-	-	-
*Snora69*	1.75	↓	8.4E-06	0.03	-	-	-
*Mmp28*	1.54	↑	8.5E-06	0.03	-	-	-
*Plscr1*	1.47	↓	1.2E-05	0.035	1.59	↓	0.017
*Deptor*	1.54	↓	1.3E-05	0.035	1.37	↓	0.029
*Ep400*	1.78	↓	1.4E-05	0.035	-	-	-
*Shroom4*	1.41	↑	1.7E-05	0.037	-	-	-
*Anp32a*	1.40	↓	1.83E-05	0.037	-	-	-
*Col16a1*	1.37	↓	1.95E-05	0.037	-	-	-
*D5Ertd579e*	1.57	↑	2.32E-05	0.039	-	-	-
*Pex5*	1.31	↑	2.35E-05	0.039	-	-	-
*A730036I17R*	1.54	↑	2.60E-05	0.039	-	-	-
*Ms4a4d*	2.01	↓	2.70E-05	0.039	-	-	-
*Prr5*	1.36	↑	2.88E-05	0.039	-	-	-
*Sdk2*	1.35	↑	3.52E-05	0.040	-	-	-
*Raldgs*	1.33	↑	5.42E-05	0.049	-	-	-
*Tinf2*	1.33	↑	5.52E-05	0.049	-	-	-
*Mir877*	1.76	↑	5.90E-05	0.050	-	-	-
*Mgp*	2.75	↓	6.31E-05	0.050	-	-	-
*Cd302*	1.43	↓	6.70E-05	0.050	-	-	-
*Snora15*	1.50	↓	6.77E-05	0.050	-	-	-
*Fam69a*	1.42	↑	6.81E-05	0.050	-	-	-

**Fig 2:**
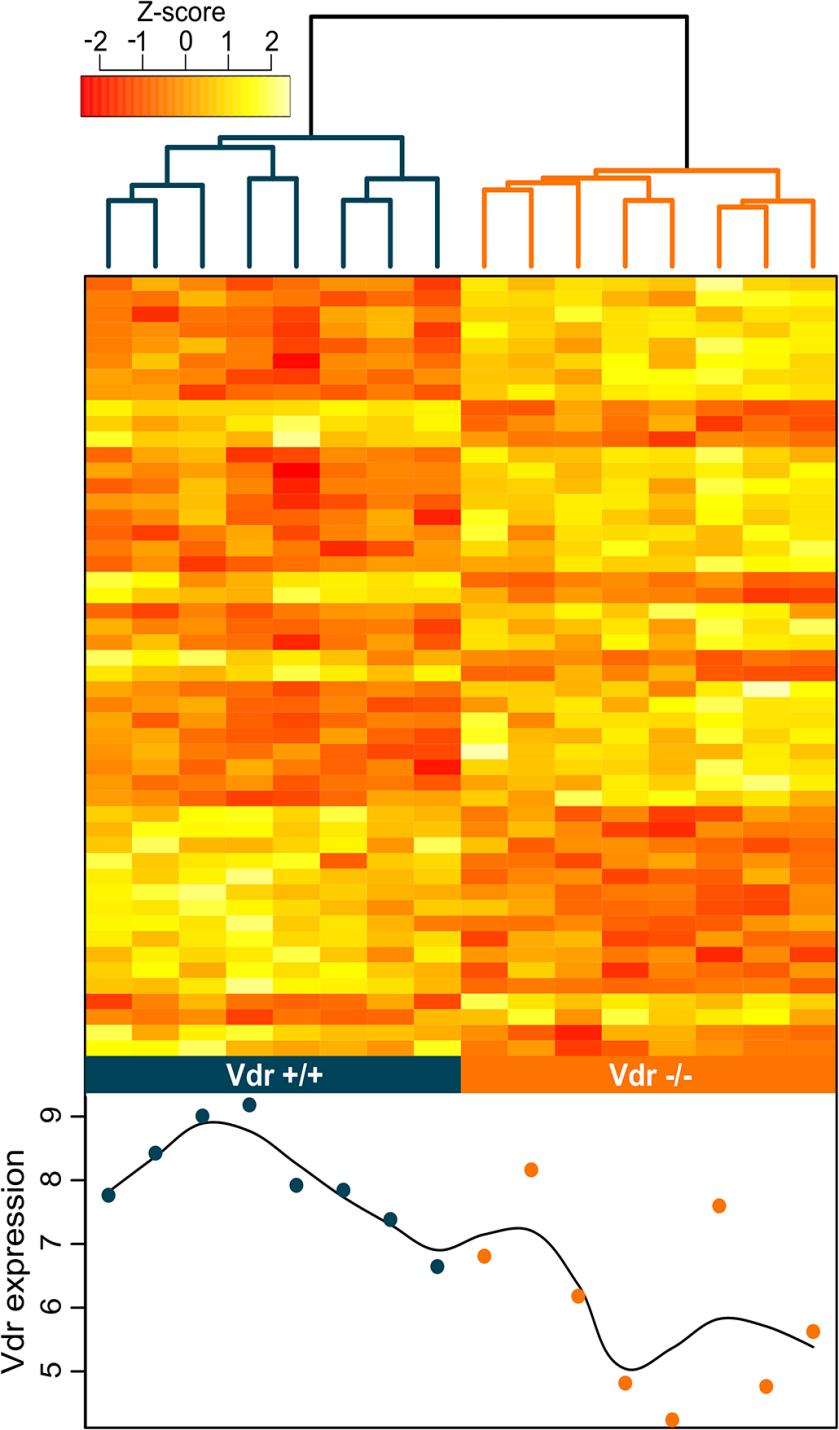
Differential gene expression between *Vdr* ^*-/-*^ and *Vdr* ^*+/+*^ placentae as determined by microarray analysis. (A) Expression differences of the top 50 differentially expressed genes in the placenta between *Vdr* ^**-/-**^ and *Vdr* ^**+/+**^ mice represented in a heatmap of z-scores. Columns represent each sample and rows represent genes. The dendrogram above the heat map shows samples cluster into genotype groups. (B) Normalised *Vdr* expression for each sample shown on dot plot below heat map. Orange points represent *Vdr* ^**-/-**^ samples, Blue points represent *Vdr* ^**+/+**^ samples.

Although only 25 genes were classed as statistically different between *Vdr* ^-/-^ and *Vdr* ^+/+^ groups, unsupervised clustering analysis of the top 50 differentially expressed genes grouped samples together by genotype, and inspection of the standardised z-scores revealed distinct patterns in gene expression between the groups with unknown subsequent effects in offspring (Fig 2A).

### Microarray validation by qPCR

**Fig 3:**
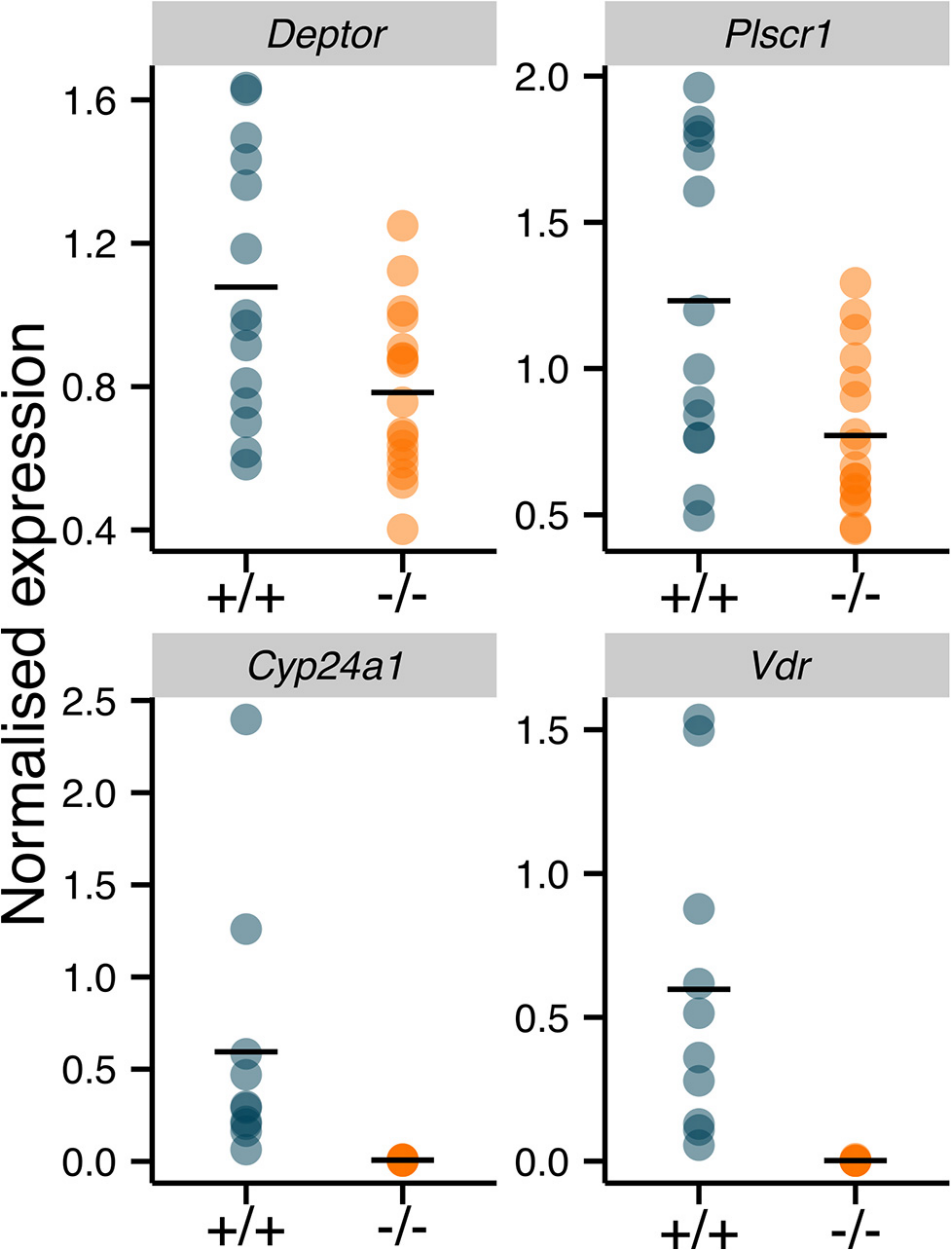
Validation of microarray differential expression by quantitative PCR. Significantly lower expression (*P*<0.05) of *Deptor*, *Plscr1*, *Cyp24a1* and *Vdr* was observed n *Vdr* ^**-/-**^ (n = 16) placentae compared to *Vdr* ^**+/+**^ (n = 17) placentae. Gene expression was normalised to *Hbms*, horizontal line represents mean.

Independent validation of the microarray results on 17 *Vdr* ^+/+^ and 16 *Vdr* ^-/-^ placentae was performed by qPCR and included additional biological replicates. *Vdr* expression was virtually undetectable in *Vdr* ^-/-^ placentae by both microarray (Fig 2B) and qPCR (Fig 3). Validation by qPCR of the microarray findings eliminates the possibility of significant transcript contamination from the heterozygous maternal tissues, as the wild type allele was not detected. Therefore, it is likely that the *Vdr* background levels of expression in the microarray data is the result of non-specific cDNA binding with the Vdr probes. Further expression analysis of *Cyp24a1*, *Deptor* and *Plscr1* by qPCR correlated with results obtained by microarray and showed that even changes <1.5 fold, such as with *Plscr1*, were replicable (Table 2 and Fig 3).

### VDRE enrichment analysis

**Fig 4:**
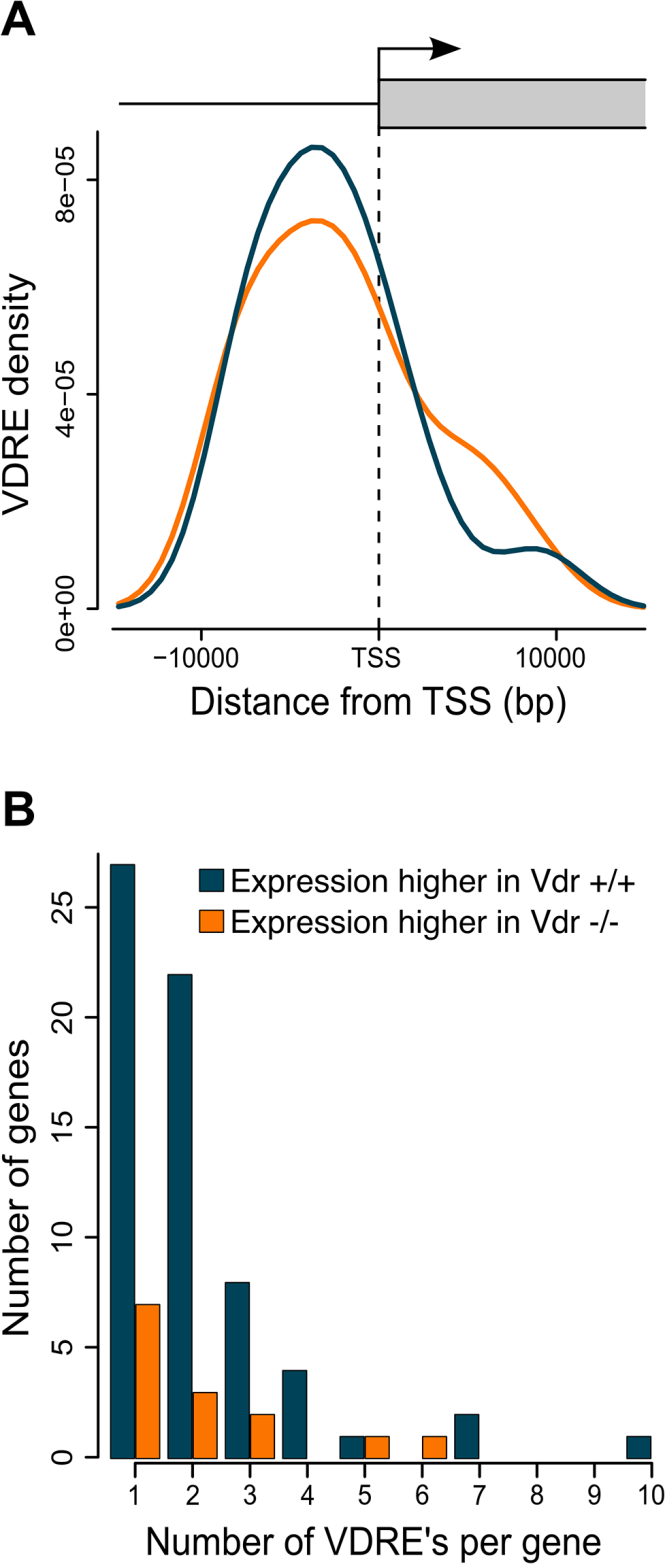
VDRE enrichment analysis of diffterentially expressed genes between *Vdr* ^-/-^ and *Vdr* ^+/+^ placentae. (A) Density of predicted VDR transcription factor binding sites in the sequence flanking transcription start sites (TSS) of genes differentially expressed between *Vdr* ^**-/-**^ and *Vdr* ^**+/+**^ samples. Blue curve represents genes more highly expressed in *Vdr* ^**+/+**^ samples, orange curve represents genes more highly expressed in *Vdr* ^**-/-**^ samples. We have used kernel density estimation to model the distribution of VDR transcription factor binding sites. (B) Number of predicted VDR binding sites per gene for genes expressed more highly in *Vdr* ^***+/+***^ placentae (blue bars) and those more highly expressed in *Vdr* ^***-/-***^ placentae (orange bars).

**Fig 5:**
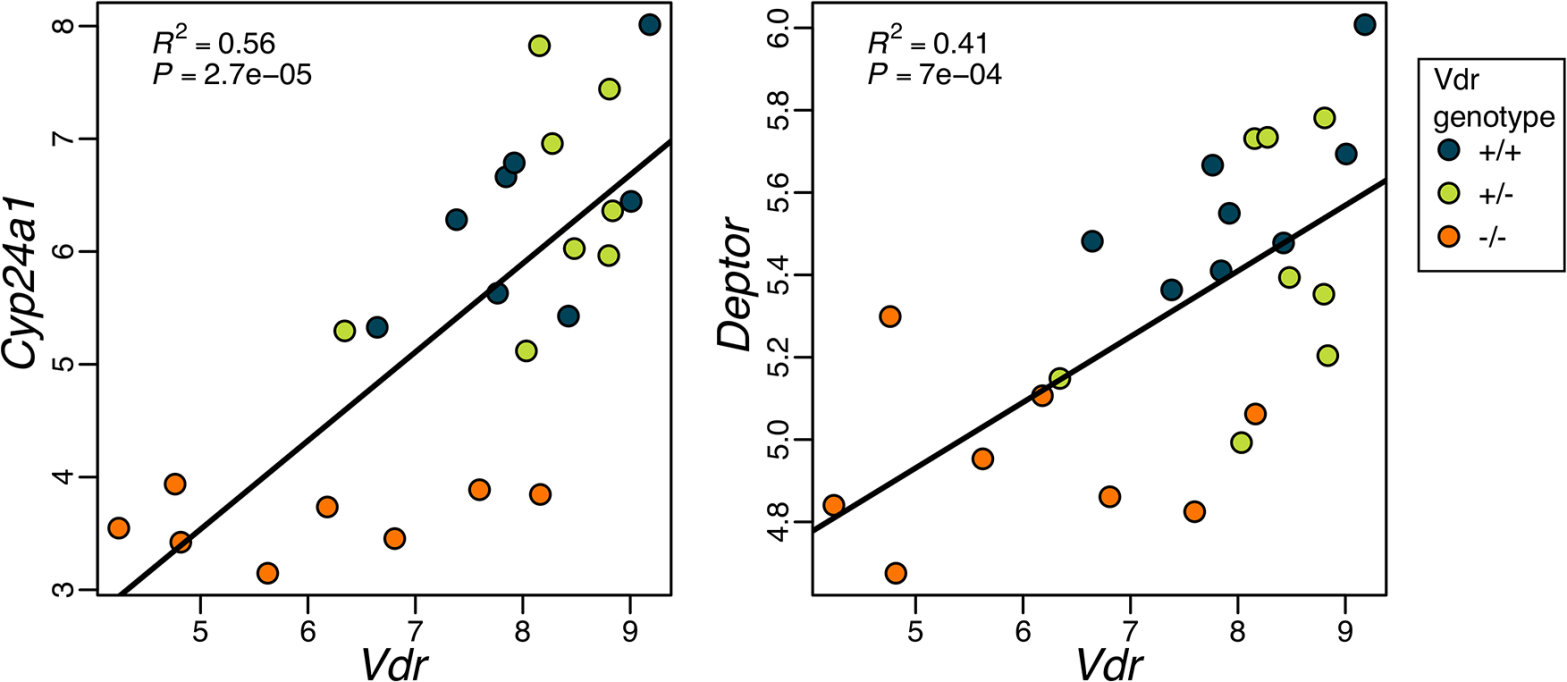
Correlations between *Vdr* expression with *Cyp24a1* and *Deptor* expression. Positive correlations of *Vdr* expression with *Cyp24a1* and *Deptor* expression in the placentae of *Vdr* ^**-/-**^, *Vdr* ^**+/-**^ and *Vdr* ^**+/+**^ mice. Individual samples are represented by colored points, black line represents linear model fit.

To assess if differential expression between *Vdr* ^+/+^ and *Vdr* ^-/-^ placentae was potentially driven by the VDR-RXR transcription factor complex, we searched for the presence of VDR-RXR transcription factor motifs in the 10kb up and down-stream of the transcription start sites of differentially expressed genes. These analyses revealed that genes that were more highly in *Vdr* ^+/+^ placentae feature more VDR binding motifs in the regions upstream of transcriptional start sites (Fig 4A), with many of these genes having more than one site per gene (Fig 4B). Expression of *Vdr* was also positively correlated with the expression of genes with upstream VDRE’s such as *Cyp24a1* (R^2^ = 0.56, *P* = 2.7e-05) and *Deptor* (R^2^ = 0.41, *P* = 7e-04) (Fig 5).

## Discussion

Although maternal vitamin D deficiency has been implicated in the pathogenesis of several pregnancy complications attributed to impaired or abnormal placental function, there are few clues indicating the mechanistic role(s) of vitamin D in their pathogenesis. Preeclampsia, preterm birth and intrauterine growth restriction have been associated with impaired placental trophoblast invasion, and remodelling, of the uterine vasculature. Vitamin D metabolites have recently been shown to enhance trophoblast invasion *in vitro* [48] and together with the presence of a local placental vitamin D metabolic pathway [22] suggest a direct role for vitamin D in the placenta. To further understand how vitamin D may influence placental development, and thereby pregnancy outcome, we used a *Vdr* gene ablated mouse model with heterozygous matings to assess placental morphological parameters and global gene expression near term without confounding by the absence of maternal vitamin D signalling. Despite analyzing multiple aspects of placental morphology including total cell volume densities, the proportion of labyrinth to junctional zones, trophoblast, fetal capillary and maternal blood space volume densities and volumes and total surface area of trophoblast cells for exchange, no differences were observed between knockout and wild type placentae. Nor were there any observed differences in fetal and placental weights indicating apparently normal function. Previous reports have found that VDR-mediated signaling in the placenta is not required for the transport of calcium to the fetus or for fetal bone mineralization in offspring born to *Vdr* ^+/-^ dams [36]. Consistent with these findings, we found no apparent phenotype in the *Vdr* ^-/-^ fetus or placenta when gestated in a heterozygous mother with adequate dietary vitamin D and calcium. In contrast, vitamin D-deficient dams carried pregnancies with smaller placentae are and reduced fetal capillary diameter [38]. Thus, the effects of maternal vitamin D deficiency on placental structure are likely mediated through the decidua rather than directly via VDR signaling in the placenta.

In profiling placental transcriptomes by microarray, we detected 25 differentially expressed genes between *Vdr* ^-/-^ and *Vdr* ^+/+^ placentae, a number of which have been shown to be expressed in the human placenta (*Tinf2* [49], *Rgsl7* [50], *Plscr1* [51] *Cd302* [52]). The greatest gene expression difference observed between *Vdr* ^-/-^ and *Vdr* ^+/+^ placentae was for *Cyp24a1* (Table 2), a gene that directly interacts with VDR in the canonical vitamin D signaling pathway and plays a key role in the vitamin D endocrine system negative feedback loop [53]. Our results show that expression of *Vdr* and *Cyp24a1* are positively correlated (Fig 4). *Cyp24a1* expression is typically induced directly by 1,25(OH)_2_D_3_ via a VDR-mediated transcriptional response [54], therefore significant reduction in *Cyp24a1* expression in *Vdr* ^-/-^ placentae was expected, and suggests a functional role for VDR in the placenta.

Expression of both *Pex5* (which encodes the peroxisome-targeting signal 1 receptor) and *Tinf2* [which encodes the TERF1-interacting nuclear factor 2 (Tin2)] was greater in *Vdr* ^-/-^ placentae when compared to *Vdr* ^+/+^. Pex5 plays a central role in the function of peroxisomes which are present in cells to clear reactive oxygen species (ROS) like hydrogen peroxide [55]. Tin2 is a component of the shelterin telomere protection complex which acts to protect telomeres from DNA damage [56] potentially caused by ROS. Increased expression of both genes within *Vdr* ^-/-^ placentae may be indicative of increased ROS, therefore increased oxidative stress, which has been hypothesized to be an underlying factor in the development of pregnancy complications like preeclampsia [57]. However, further work is required in order to establish whether there is an over-production of oxidative species within *Vdr* ^-/-^ placentae.

Of particular interest, we observed lower *Deptor* expression in *Vdr* ^-/-^ placentae and higher expression of *Prr5* when compared to *Vdr* ^+/+^. Both genes are components of the mTOR signaling pathway. During pregnancy, placental mTOR signaling plays an important role in the regulation of fetal growth, particularly as a maternal nutrient and growth factor sensor [58]. Furthermore, both *DEPTOR* and *PRR5* have been shown to be highly expressed within the placenta [59, 60]. Deptor is an inhibitor of the mTOR signaling pathway [61] and by directly binding to mTORC1 and mTORC2 it acts to inhibit cell proliferation and protein synthesis. Alternately, *Prr5* is a component of the mTORC2 complex that promotes cell growth through its interaction with Rictor [62]. It has been hypothesized that 1,25(OH)_2_D, through VDR signaling, can suppress downstream mTOR signaling [63]. In *Vdr* ^-/-^ placentae decreased *Deptor* and increased *Prr5* indicates activation of the mTOR pathway. Thus, mTOR activation may explain why there was no difference in fetal weight and placental structure as there would be a drive for growth which may normalize any differences between the genotypes (Fig 1).

Our results indicate that VDR signaling in the placenta is not essential for pregnancy success. This is supported by recent studies assessing placental VDR expression and polymorphisms in complicated pregnancies. VDR polymorphisms do not appear to predispose women to preeclampsia and gestational hypertension [64] and VDR expression is similar between normal placentae and those from pregnancies complicated by gestational diabetes [65]. Furthermore, there is no linear correlation between placental VDR protein expression and birth weight [66]. Cho *et al*. did, however, observe that 85% of women suffering gestational diabetes were classified as vitamin D deficient (25(OH)D serum level <20 ng/mL)[65]. Maternal vitamin D deficiency has been associated with pregnancy complications such as preeclampsia, small for gestational age and preterm birth [67] suggesting important roles for vitamin D in maternal tissues.

In this study, we used *Vdr* ^+/-^ dams to assess the effect of vitamin D on placental and fetal development without the confounding factor of poor maternal health seen in *Vdr* ^-/-^ mice [39]. Despite gene expression differences between knockout and wild-type placentae, this did not translate to differences in placental morphology and function with no apparent differences in fetal outcome near term. Our results suggest that maternal vitamin D status may be more crucial in determining pregnancy outcome than VDR signaling in the conceptus alone. This may be due to the presence of non-genomic VDR signaling which has been largely ignored in many studies, as well as genomic signaling in maternal tissues including the decidua. We suggest experiments using homozygous knockout dams will need to be undertaken in order to fully investigate the potential cross-talk between the maternal decidua and the placenta in regards to VDR signaling. Furthermore, the gene expression differences observed in this study suggest some genes harbour VDRE’s in the placenta (Fig 1B and 1C) highlighting the need for further work to elucidate the role of the vitamin D endocrine pathway in placental function.

## Supporting Information

S1 File Primers and cycling conditions for PCR analyses. *Vdr* genotyping PCR primers and conditions [41] (Table A). Genotyping of Vdr alleles by PCR and gel electrophoresis for wild-type allele (Figure Aa) and for knockout allele (Figure Ab). Primers and PCR conditions for sex typing of mice [42] (Table B). PCR primers and cycling conditions to validate DNAse treatment of placental RNA extracts (Table C). Quantitative PCR assay and cycling conditions for microarray validation (Table D).
(DOCX)

S2 File Analysis methods and code for the microarray differential expression experiment. (HTML)
