# Supplementary material for: Vitamin D Receptor Gene Ablation in the Conceptus Has Limited Effects on Placental Morphology, Function and Pregnancy Outcome
Source: PLoS One. 2015 Jun 29;10(6):e0131287. doi: 10.1371/journal.pone.0131287 (PMC4488298; doi:10.1371/journal.pone.0131287)
Supplement: S1 File — Vdr genotyping PCR primers and conditions [41] (Table A). Genotyping of Vdr alleles by PCR and gel electrophoresis for wild-type allele (Figure Aa) and for knockout allele (Figure Ab). Primers and PCR conditions for sex typing of mice [42] (Table B). PCR primers and cycling conditions to validate DNAse treatment of placental RNA extracts (Table C). Quantitative PCR assay and cycling conditions for microarray validation (Table D). (DOCX) [file pone.0131287.s001.docx]

**Supplementary Data**

**Table S1.** *Vdr* genotyping PCR conditions. Panda et al. 2004 (29)

| **Wild Type *Vdr* Primers** | | | **Amplicon length** |
| --- | --- | --- | --- |
| Forward | 5’-CTCCATCCCCATGTGTCTTT-3’ | | 750 bp |
| Reverse | 5’-TTCTTCAGTGGCCAGCTCTT-3’ | |  |
| **Mutant *Vdr* Primers** | | | **Amplicon length** |
| Forward | 5’-GCTGCTCTGATGCCGCCGTGTTC-3’ | | 294 bp |
| Reverse | 5’-GCACTTCGCCCAATAGCAGCCAG-3’ | |  |
| **Cycling Conditions** | | |  |
| Denature | 94°C | 60 sec | 35 cycles |
| Anneal | 65°C | 60 sec |  |
| Extend | 72°C | 60 sec |  |

| **A** | **B** |
| --- | --- |
| 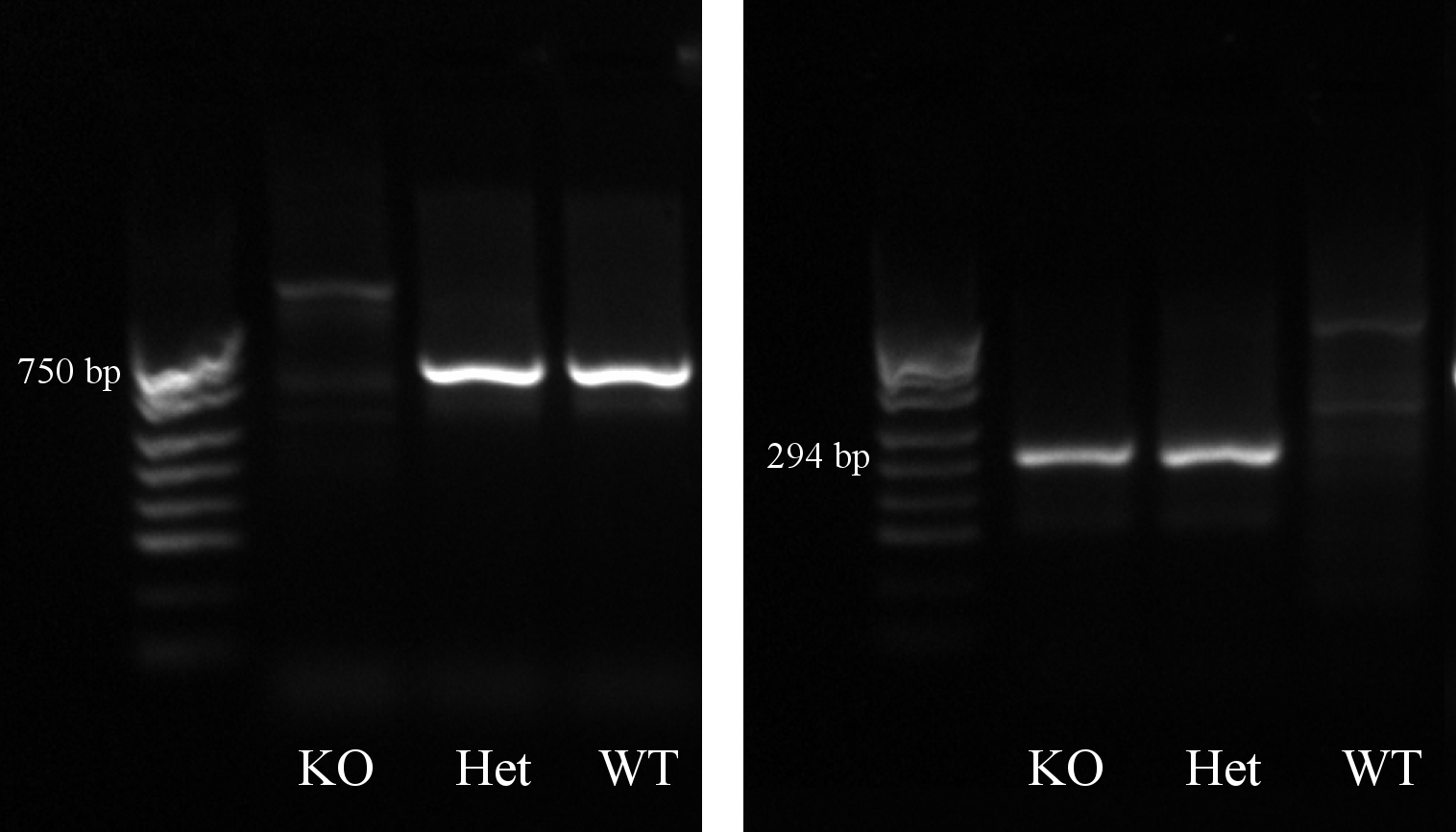 | |

**Figure S1.** Genotyping of *Vdr* alleles by PCR and Gel electrophoresis.

**A** Primers for wild-type allele. **B** Primers for knockout allele.

**Table S2.** Primers and PCR conditions for sex typing of mice. Albay et al., 2009 (30)

| ***Sry* Primers** | | | | **Amplicon length** |
| --- | --- | --- | --- | --- |
| Forward | 5’-AACAACTGGGCTTTGCACATTG-3’ | | | 7166, 146 bp (doublet) |
| Reverse | 5’-GTTTATCAGGGTTTCTCTCTAGC-3’ | | |  |
| ***Nfiα* Primers** | | | | **Amplicon length** |
| Forward | 5’-TGCTGTGTTCTGGTCAGTCAAG-3’ | | | 405 bp |
| Reverse | 5’-CAAAGCAAATCTCCATGCTCGG-3’ | | |  |
| **Cycling Conditions** | | | |  |
| Denature | 94°C | 60 sec | 33 cycles | |
| Anneal | 60°C | 60 sec |  |  |
| Extend | 72°C | 72 sec |  |  |
|  | 72°C | 9 min | 1 cycle | |

**Table S3.** PCR primers and cycling conditions to validate DNAse treatment of placental RNA extracts

| **Primers** | | | | **Amplicon length** |
| --- | --- | --- | --- | --- |
| Forward | 3’-GGCACTGACTGAGGTCAAAC-5’ | | | 120 bp |
| Reverse | 3’-GTCACAATCACAGAGACTTTGA-5’ | | |  |
| **Cycling Conditions** | | | |  |
| Denature | 94°C | 10 sec | 40 cycles | |
| Anneal | 60°C | 15 sec |  |  |
| Extend | 72°C | 60 sec |  |  |

**Table S3.** Quantitative PCR assay and cycling conditions

| **Gene** | **Taqman Assay ID** | | **Amplicon size** |
| --- | --- | --- | --- |
| *Vdr* | Mm00437297_m1 | | 95 bp |
| *Cyp24a1* | Mm00487244_m1 | | 99 bp |
| *Deptor* | Mm01195336_m1 | | 82 bp |
| *Hbms* | Mm01143545_m1 | | 81 bp |
| *Plscr1* | Mm01228223_g1 | | 79 bp |
| **Cycling Conditions** | | | |
| **Activation** | 95°C | 10 min |  |
| **Denature** | 95°C | 15 sec | 40 cycles |
| **Anneal/extend** | 60°C | 60 sec |  |
