# Supplementary material for: Vitamin D Receptor Gene Ablation in the Conceptus Has Limited Effects on Placental Morphology, Function and Pregnancy Outcome
Source: PLoS One. 2015 Jun 29;10(6):e0131287. doi: 10.1371/journal.pone.0131287 (PMC4488298; doi:10.1371/journal.pone.0131287)
Supplement: S2 File — (HTML) [file pone.0131287.s002.html]

S2 File. Analysis methods and code for the microarray differential expression experiment 


# S2 File. Analysis methods and code for the microarray differential expression experiment

All raw gene expression data from this expreiment was deposited into
the NCBI GEO repository under the accession GSE61583. The differential
expression analysis in the manuscript can be fully reproduced by using
the R code detailed below.

Set the working directory for this analysis:

### Load the required R libraries

```
library(GEOquery)
library(oligo)
library(limma)
library(arrayQualityMetrics)
library(annotate)
library(R2HTML)
library(pd.mogene.2.1.st)
library(mogene21sttranscriptcluster.db)
```

### Download the data from NCBI GEO

First, create a directory for the raw data in the working directory and download the .CEL files:

```
dir.create(path="RawData")
download.file(url="http://www.ncbi.nlm.nih.gov/geo/download/?acc=GSE61583&format=file&token=azankogknpsphox", destfile="RawData/GSE61583.tar")

untar(tarfile="RawData/GSE61583.tar", exdir="RawData/")
```

Then download the meta-data read into the current session:

```
download.file(url="http://www.ncbi.nlm.nih.gov/geo/query/acc.cgi?acc=GSE61583&targ=gsm&form=text&view=brief&token=azankogknpsphox", destfile="RawData/sampleData.soft")

sampleDat <- getGEO(filename="RawData/sampleData.soft")

# Just select the sample titles as these contain the meta data we need
sampleDat <- Meta(sampleDat)

#ID's
gsm <- sampleDat$geo_accession

# Get the sample meta data
meta <- sampleDat$characteristics_ch1

# extract genotype information
genotype <- substring(grep(pattern="genotype/variation of offspring: ", x=meta, value=TRUE), first=34)

# Change genotypes to abbreviations
genotype <- gsub(pattern="Vdr knockout", replacement="KO", x=genotype)
genotype <- gsub(pattern="Vdr heterozygous", replacement="Het", x=genotype)
genotype <- gsub(pattern="wild-type", replacement="WT", x=genotype)
head(genotype)
```

```
## [1] "WT"  "Het" "WT"  "Het" "KO"  "Het"
```

```
# extract the litter information
litter <- substring(grep(pattern="litter_id:", x=meta, value=TRUE), first=12)

# Create phenodata data frame
pData <- data.frame(Genotype = genotype, Litter = litter)
row.names(pData) <- gsm
head(pData)
```

```
##            Genotype Litter
## GSM1508835       WT    108
## GSM1508836      Het     12
## GSM1508837       WT     14
## GSM1508838      Het     14
## GSM1508839       KO     14
## GSM1508840      Het     21
```

### Import, preprocess and normalise the raw array data

Load the .CEL files for processing:

```
celFiles <- list.celfiles(path="rawData/", full.names=TRUE, listGzipped=TRUE)
affyExpressionFS <- read.celfiles(celFiles)
```

Normalise the arrays using the rma method with background
subtraction. The rma normalisation is performed at the individual probe
level. This function will return the data in the expressionSet class.

```
eset <- rma(affyExpressionFS, normalize=T, background=T)

# remove the file name extensions from the sample names, so the GEO records ID's are the sample names
sampleNames(eset) <- substring(sampleNames(eset), first=1, last=10)

# check the dimensions and sample names of expressionSet
dim(eset)
head(sampleNames(eset))
```

### Add sample phenotype and annotation information

Check that the phenodata table created earlier matches the expressionSet data for the order of samples. This test must return `TRUE`:

```
all(rownames(pData) == colnames(eset))
```

```
## [1] TRUE
```

Add the sample information to the phenodata slot of the expressionSet:

```
pData(eset) <- pData
```

Add annotation data to the expressionSet. This includes gene symbol, Ensembl gene ID and chromosomal locations.

```
# Get the transcript cluster IDs from the expressionset
ID <- featureNames(eset)

# Look up the Gene Symbol, name, and Ensembl Gene ID for each of those IDs
Symbol <- getSYMBOL(ID, "mogene21sttranscriptcluster.db")
Name <- as.character(lookUp(ID, "mogene21sttranscriptcluster.db",
                            "GENENAME"))
Ensembl <- as.character(lookUp(ID, "mogene21sttranscriptcluster.db",
                               "ENSEMBL"))
Chr <- as.character(lookUp(ID, "mogene21sttranscriptcluster.db", "CHR"))
Start <- as.character(lookUp(ID, "mogene21sttranscriptcluster.db", "CHRLOC"))
End <- as.character(lookUp(ID, "mogene21sttranscriptcluster.db", "CHRLOCEND"))

# Add hyperlink info for Ensembl ID's
Ensembl <- ifelse(Ensembl=="NA", NA, 
                  paste("<a href='http://asia.ensembl.org/Mus_musculus/Gene/Summary?g=", Ensembl, "'>", Ensembl, "</a>", sep=""))

# Make a data frame with all those identifiers
tmp <- data.frame(ID=ID, Symbol=Symbol, Name=Name, Ensembl=Ensembl, Chr=Chr,
                  Start=Start, End=End, stringsAsFactors=F)

# The stringsAsFactors makes "NA" characters. This fixes that problem.
tmp[tmp=="NA"] <- NA

# set the featureData of the expressionSet using the data frame created above.
fData(eset) <- tmp
```

Count the number of probes with no annotation information:

```
sum(is.na(fData(eset)$Symbol))
```

```
## [1] 16636
```

We see there are many probes that do not have annotation information
and are subsequently of little use for the downstream analyses. These
include control probes and those withot a good match to any genes or
have low specificity.

Remove these un-annotated probes from the expressionSet:

```
dim(eset)
```

```
## Features  Samples 
##    41345       24
```

```
eset <- eset[!is.na(fData(eset)$Symbol), ]
dim(eset)
```

```
## Features  Samples 
##    24709       24
```

Exoprt table of normalised expression values:

```
normDat <- exprs(eset)
ID_REF <- row.names(normDat)
normDat <- cbind(ID_REF, normDat[ ,1:24])
row.names(normDat) <- NULL
dir.create(path="processedData")
write.table(x=normDat, file="processedData/normalisedData.txt", row.names=FALSE, col.names=TRUE, quote=F, sep="\t")
```

### Differential expression analysis

Differential expression analysis will compare genotype groups and include the litters as a blocking factor.

First, the comparison matrix is set up and the correlation coefficient between samples is calculated.

```
# Set up analysis matrix
Treat <- factor(pData(eset)[ ,1], levels=c("WT", "Het", "KO"))
design <- model.matrix(~0+Treat)
colnames(design) <- levels(Treat)

# Estimate the correlation between measurements made for each Litter
# Warning: This computation can be time consuming 
corfit <- duplicateCorrelation(eset, design, block=pData(eset)$Litter)
corfit$consensus.correlation
```

Then the linear model is fitted and the contrasts between groups is made:

```
fit <- lmFit(eset, design, block=pData(eset)$Litter,
             correlation=corfit$consensus.correlation)

contrast.matrix <- makeContrasts(WT-KO, WT-Het, Het-KO, levels = design)

fit2 <- contrasts.fit(fit, contrast.matrix)
fit2 <- eBayes(fit2)
```

Show the number of significant genes defined as absolute fold-change > 1.3 and FDR < 0.05.

```
results <- decideTests(fit2, adjust.method="fdr", p.value=0.05,
                       lfc=abs(log2(1.3)))

vennDiagram(results, circle.col=c("#004358", "#FD7400", "Black"),
            cex=c(1, 1, 1))
```

Have a look at the top genes for the wild-type vs knockout comaprison:

```
topTable(fit2, coef="WT - KO")[ ,c(2, 8:11)]
```

```
##            Symbol   logFC AveExpr      t   P.Value
## 17394965  Cyp24a1  2.6938   5.439  7.630 5.019e-08
## 17316348 Snord123  0.6551   7.033  5.815 4.368e-06
## 17216070    Atg4b -0.4092   8.057 -5.805 4.478e-06
## 17506356    Zfpm1 -0.3506   8.374 -5.736 5.341e-06
## 17279167  Snora28  0.5736   7.830  5.725 5.502e-06
## 17541008  Snora69  0.8017   7.264  5.562 8.353e-06
## 17266911    Mmp28 -0.6188   6.099 -5.557 8.481e-06
## 17520315   Plscr1  0.5514   4.948  5.433 1.167e-05
## 17547877   Deptor  0.6256   5.317  5.395 1.285e-05
## 17451126    Ep400  0.8285   8.028  5.354 1.433e-05
```

Create data frames containing the results for each contrast and export the top 500 to HTML files:

```
WT.KO <- topTable(fit2, coef="WT - KO", number=length(featureNames(eset)))
WT.Het <- topTable(fit2, coef="WT - Het", number=length(featureNames(eset)))
Het.KO <- topTable(fit2, coef="Het - KO", number=length(featureNames(eset)))


HTML(WT.KO[1:500, ], file="processedData/WT-KO.html", append=FALSE)
HTML(WT.Het[1:500, ], file="processedData/WT-Het.html", append=FALSE)
HTML(Het.KO[1:500, ], file="processedData/Het-KO.html", append=FALSE)
```

Details of all the packages used in this analysis:

```
sessionInfo()
```

```
## R version 3.1.0 (2014-04-10)
## Platform: x86_64-apple-darwin13.1.0 (64-bit)
## 
## locale:
## [1] en_AU.UTF-8/en_AU.UTF-8/en_AU.UTF-8/C/en_AU.UTF-8/en_AU.UTF-8
## 
## attached base packages:
## [1] parallel  stats     graphics  grDevices utils     datasets  methods  
## [8] base     
## 
## other attached packages:
##  [1] statmod_1.4.20                       
##  [2] R2HTML_2.3.0                         
##  [3] pd.mogene.2.1.st_2.14.0              
##  [4] oligo_1.28.2                         
##  [5] oligoClasses_1.26.0                  
##  [6] mogene21sttranscriptcluster.db_2.14.0
##  [7] org.Mm.eg.db_2.14.0                  
##  [8] RSQLite_0.11.4                       
##  [9] limma_3.20.9                         
## [10] GEOquery_2.30.1                      
## [11] DBI_0.3.0                            
## [12] Biostrings_2.32.1                    
## [13] XVector_0.4.0                        
## [14] IRanges_1.22.10                      
## [15] arrayQualityMetrics_3.20.0           
## [16] annotate_1.42.1                      
## [17] AnnotationDbi_1.26.0                 
## [18] GenomeInfoDb_1.0.2                   
## [19] Biobase_2.24.0                       
## [20] BiocGenerics_0.10.0                  
## [21] knitr_1.6                            
## 
## loaded via a namespace (and not attached):
##  [1] acepack_1.3-3.3       affxparser_1.36.0     affy_1.42.3          
##  [4] affyio_1.32.0         affyPLM_1.40.1        base64_1.1           
##  [7] beadarray_2.14.1      BeadDataPackR_1.16.0  BiocInstaller_1.14.2 
## [10] bit_1.1-12            Cairo_1.5-6           cluster_1.15.3       
## [13] codetools_0.2-9       colorspace_1.2-4      digest_0.6.4         
## [16] evaluate_0.5.5        ff_2.2-13             foreach_1.4.2        
## [19] foreign_0.8-61        formatR_1.0           Formula_1.1-2        
## [22] gcrma_2.36.0          genefilter_1.46.1     GenomicRanges_1.16.4 
## [25] grid_3.1.0            gridSVG_1.4-0         Hmisc_3.14-5         
## [28] hwriter_1.3.2         illuminaio_0.6.0      iterators_1.0.7      
## [31] lattice_0.20-29       latticeExtra_0.6-26   nnet_7.3-8           
## [34] plyr_1.8.1            preprocessCore_1.26.1 RColorBrewer_1.0-5   
## [37] Rcpp_0.11.2           RCurl_1.95-4.3        reshape2_1.4         
## [40] RJSONIO_1.3-0         rpart_4.1-8           setRNG_2011.11-2     
## [43] splines_3.1.0         stats4_3.1.0          stringr_0.6.2        
## [46] survival_2.37-7       SVGAnnotation_0.93-1  tools_3.1.0          
## [49] vsn_3.32.0            XML_3.98-1.1          xtable_1.7-4         
## [52] zlibbioc_1.10.0
```
